# Generation Scotland – Linking all the records we can

**DOI:** 10.23889/ijpds.v9i5.2862

**Published:** 2024-09-18

**Authors:** Archie Campbell, Robin Flaig, Daniel McCartney, Heather Whalley, Cathie Sudlow

**Affiliations:** 1 University of Edinburgh; 2 Health Data Research UK

## Objectives

Generation Scotland (GS) is a family-based genetic epidemiology study. Initial recruitment between 2006-11 recruited ~24,000 adults from ~7000 families across Scotland with consent for medical record linkage and re-contact. In 2022 we began recruiting another 20,000, with consent extended to administrative records, with age range now 12+.

## Methods

Original volunteers completed a baseline questionnaire, provided biological samples and underwent clinical assessment. The samples, phenotype and genotype (including methylation) data are linked to routine NHS hospital, maternity, lab test, prescriptions, dentistry, mortality, imaging, cancer screening, GP data records, Covid-19 testing and vaccinations, and follow-up questionnaires. The new wave of recruitment is all online and can be done on a smartphone, with DNA from saliva collected by post. Teenagers aged 12-15 can join with parental consent.

## Results

Researchers can find prevalent and incident disease cases and controls, to test research hypotheses on a stratified population. GS is a vanguard cohort testing emerging linkage opportunities in Scotland – including neonatal bloodspots and medical imaging reports. GS has established and validated E-HR linkage with the NHS Scotland CHI Register, overcoming technical and governance issues in the process. We contribute to major international consortia, with collaborators from institutions worldwide, both academic and commercial.

## Conclusion

We plan to extend the linkage process to include other administrative data from national datasets as and when approvals are obtained. New types of data can also be collected by online questionnaires. The Research Tissue Bank resources are available to academic and commercial researchers through a managed access process.

